# Analyzes of genome-wide association follow-up study for calving traits in dairy cattle

**DOI:** 10.1186/1471-2156-13-71

**Published:** 2012-08-14

**Authors:** Johanna K Höglund, Bernt Guldbrandtsen, Mogens S Lund, Goutam Sahana

**Affiliations:** 1 Department of Molecular Biology and Genetics, Aarhus University, P.O. Box 50, Tjele, DK-8830, Denmark; 2 VikingGenetics, Ebeltoftvej 16, Assentoft, Randers, SØ, DK-8960, Denmark; 3 Department of Animal Breeding and Genetics, Swedish University of Agricultural Sciences, P.O. Box 7070, Uppsala, 750 07, Sweden

**Keywords:** Association mapping, Confirmation study, Calving traits, QTL, Cattle

## Abstract

**Background:**

There is often a pronounced disagreement between results obtained from different genome-wide association studies in cattle. There are multiple reasons for this disagreement. Particularly the presence of false positives leads to a need to validate detected QTL before they are optimally incorporated or weighted in selection decisions or further studied for causal gene. In dairy cattle progeny testing scheme new data is routinely accumulated which can be used to validate previously discovered associations. However, the data is not an independent sample and the sample size may not be sufficient to have enough power to validate previous discoveries. Here we compared two strategies to validate previously detected QTL when new data is added from the same study population. We compare analyzing a combined dataset (COMB) including all data presently available to only analyzing a validation dataset (VAL) i.e. a new dataset not previously analyzed as an independent replication. Secondly, we confirm SNP detected in the Reference population (REF) (i.e. previously analyzed dataset consists of older bulls) in the VAL dataset.

**Results:**

Clearly the results from the combined (COMB) dataset which had nearly twice the sample size of other two subsets allowed the detection of far more significant associations than the two smaller subsets. The number of significant SNPs in REF (older bulls) was about four times higher compare to VAL (younger bulls) though both had similar sample sizes, 2,219 and 2,039 respectively. A total of 424 SNP-trait combinations on 22 chromosomes showed genome-wide significant association involving 284 unique SNPs in the COMB dataset. In the REF data set 101 associations (73 unique SNPs) and in the VAL 24 associations (18 unique SNPs) were found genome-wide significant. Sixty-eight percent of the SNPs in the REF dataset could be confirmed in the VAL dataset. Out of 469 unique SNPs showing chromosome-wide significant association with calving traits in the REF dataset 321 could be confirmed in the VAL dataset at P < 0.05.

**Conclusions:**

The follow-up study for GWAS in cattle will depend on the aim of the study. If the aim is to discover novel QTL, analyses of the COMB dataset is recommended, while in case of identification of the causal mutation underlying a QTL, confirmation of the discovered SNPs are necessary to avoid following a false positive.

## Background

Sample sizes in the thousands are generally required for genome-wide association studies (GWAS) to have sufficient statistical power to detect moderate sized associations [1]. The large sample sizes and initial high cost of SNP arrays helped motivate the development and use of multistage GWA study designs [2]. Replication in association studies is necessary because there is often a pronounced disagreement between studies in the results obtained. Therefore, it is necessary to validate detected associations before they are optimally incorporated or weighted in selection decisions or large sums are invested into identification of causal factors.

Within the Holstein cattle population it is impossible to conduct a genuinely independent GWAS confirmation study following guidelines from NCI-NHGRI Working Group on Replication in Association Studies [3] due to unavailability of unrelated samples. The effective population size in Holstein cattle is about 50 [4]. Thus any QTL study conducted in Holstein will par force include animals that are closely related to the study population of any other study conducted in Holstein cattle. Nevertheless, one is still able to validate QTL detected in different studies, as detection of QTL in the same region from different samples of the population strengthens evidence for the QTL being real.

In dairy cattle breeding, new data is routinely recorded. This new data provides an opportunity to validate earlier detected QTL. When new data are obtained from the same population there are two choices: either the analyses of new data are considered as an independent replication to confirm the findings from the previous study or they are treated as additional information used to fortify the conclusions through an analysis of a combined dataset. However, both approaches have their own limitations. In the first approach we assume that the new data is an independent sample which is not correct because the animals are related through the pedigree. In the second approach, analyzing a combined dataset uses the information in the reference dataset twice. Thus information in the reference data is used both in the discovery of QTL as well as in their subsequent confirmation. This makes interpretation as a confirmation study difficult [5].

A genome-wide association study for calving traits has been conducted for the combined Danish and Swedish populations of Holstein cattle [6]. To this end we obtained substantial new data from the same study population as was analyzed in the previous study. In this study we wanted to compare the performance of two approaches, to evaluate QTL detected in a follow-up study. We compare analyzing the combined dataset (COMB) to only analyzing the new data alone and try to confirm SNPs which showed significant association with traits in the reference (REF) dataset to the validation (VAL) dataset. The first objective of the present study was to evaluate the performance of strategies for follow-up association studies in cattle. Another objective of this study was to map QTL for calving traits segregating in the Nordic Holstein cattle using the full combined dataset. As sample size was substantially higher in the COMB dataset compared to the REF, it was expected that COMB will have higher power to detect novel QTL than either of the two subsets.

## Methods

### Animals and Phenotypes

A total of 4,258 bulls were available for the study. Of these data 2,219 were previously analyzed by Sahana et al. [6]. These animals constitute the reference population. The validation population consisted of 2,039 new bulls those were added as the genotypes and breeding values for calving traits from these bulls had subsequently become available. Single-trait breeding values (**STBV**) were predicted for each animal using best linear unbiased prediction procedures and a sire model where sires were treated as unrelated. Pedigree information was not included in prediction of STBV to avoid the risk that SNP would be selected on the basis of pedigree information rather than phenotype. Thus the STBV of a sire is predicted from its daughters’ information only. These STBVs were generated specifically for QTL mapping studies by the Nordic Cattle Genetic Evaluation company (http://www.nordicebv.info) in order to avoid identification of false positive associations from QTL affecting correlated traits. STBV were estimated using the models described by Danish Cattle Federation [7] simultaneously incorporating direct and maternal additive genetic effects. STBV values were estimated based on recordings undertaken as part of the routine recording system of Denmark, Sweden and Finland. Two STBVs were predicted for each animal: one for 1^st^ parity, and the other for combined 2^nd^ and later (up to fifth) parities. Data were edited according to national editing rules, including removal of twin pregnancies, crossbred pregnancies and pregnancies resulting from embryo transfer. Separate STBVs were calculated for direct and maternal effects. The direct effect represents the additive effect of the genotype of the calf being born. The maternal effect represents the additive genetic effect of the genotype of the cow giving birth. STBV were standardized to an average of 100 and a standard deviation of 10 index units, with standardization factors calculated from the sire population born 1997–1998. The data recording systems and breeding value estimation for calving traits were described previously [8]. Additional details about the traits and method of estimation of breeding values is available at the website of Nordic Cattle Genetic Evaluation http://www.nordicebv.info/Forside.htm.

The traits analyzed were calving ease (**CE**), calf size (**CS**) and stillbirth (**SB**). The registrations of phenotype for these traits were previously described by Sahana et al. [6]. For each of these three recorded traits four single trait breeding values (STBV) were calculated: one for each combination of direct (**D**) and maternal (**M**) effect, and first (**F**) and later (**L**) pregnancy. For example the STBV for a direct genetic effect for stillbirth in 2^nd^ and later lactations is designated as DSBL. Additionally, two combined indices were analyzed. The birth index (**BI**) is a compound index describing a sire’s total direct additive genetic effect on calving ease by combining DSBF, DSBL, DCEF, DCEL, DCSF and DCSL. Likewise the calving index (**CI**) is a compound index describing the maternal additive genetic effect on calving ease by combining the equivalent maternal STBV. The sub-traits are combined using an economically weighted average (for economic weights, see Pedersen et al.[9]).

The analyses were done for three datasets. The reference dataset (REF) was the data of the bulls analyzed by Sahana et al. [6]. A set of 2,039 new bulls with phenotypes and markers types not included in the previous study of Sahana et al. [6] was used for validation of association (VAL). The third set was combined data of 4,258 bulls (COMB). The numbers of phenotypes available for analyses for each trait for all the three datasets are presented in Table 1.

**Table 1:** Number of sire phenotypes available for analyses for calving traits for three datasets

**Trait**^**#**^	**REF**	**VAL**	**COMB**
BI	2219	2039	4258
CI	2219	1348	3567
DCEF	1367	1629	2996
DCEL	2214	2007	4221
DSBF	1505	1698	3203
DSBL	2214	2009	4223
DCSF	1155	1110	2265
DCSL	2034	1525	3559
MCEF	2207	1266	3473
MCEL	2114	973	3087
MSBF	2210	1268	3478
MSBL	2129	981	3110
MCEF	2029	943	2972
MCEL	1916	702	2618

The reference dataset (REF) was previously analyzed; the validation population (VAL) is the new record accumulated and the combined (COMB) is all the records available (i.e. REF + VAL).

^#^Prefixes: D = direct effect; M = maternal effect. Calving traits: BI = birth index; SB = stillbirth; CE = calving ease; CS = calf survival; CI = calving index. Suffixes: F = first lactation; L = later lactation.

### SNP Chip and Genotyping

We used the BovineSNP50 Beadchip (Illumina, Inc., San Digeo, CA, USA) to genotype 4,258 animals (for details on SNP genotyping, see Sahana et al. [6]). This assay includes 54,001 markers with a median interval of 37 kb between SNPs. SNPs with a minor allelic frequency (**MAF**) of less than 5%, or with call rates less than 95% were excluded. Likewise, individuals with call rates less than 85%, or average GC scores of less than 0.65 were excluded. The minimal accepted GC score for individual typings was 0.60. After editing, the final marker set included 38,545 SNPs on 29 bovine autosomes (**BTA**). The number of SNPs included for analysis varied between 2,502 on BTA1 and 724 on BTA28. The SNP positions within a chromosome were based on the University of Maryland assembly UMD3.1 (http://www.cbcb.umd.edu/research/bos_taurus_assembly.shtml). For comparison of physical locations of the markers from previously reported studies, markers were mapped to the University of Maryland assembly UMD3.1, which enables direct comparison.

### Statistical Methods for Association Analysis

The Analyses were done using the following linear model

(1) $$y_{i}=\mu +bx_{i}+s_{i}+e_{i}$$

where y_i_ is the estimated breeding value of individual i for the trait, μ is a shared fixed effect, x_i_ is a count in individual i of one of the two alleles (with an arbitrary labeling of alleles), b is the fixed allele substitution effect, s_i_ is the fixed effect of the sire of individual i and e_i_ is a random residual of individual i assumed to a normal distribution with mean zero and unknown variance. Testing was done by a Wald test with a null hypothesis of H_0_: b = 0. The analyses were done in software R (http://www.r-project.org).

### Identifying and validating significant SNP markers

The significant thresholds (i.e. p-value) chosen for identifying significant association is important for the outcome. We used two different approaches for identify significant SNP, one for the discovery of QTL and another for validating discovered associations. For QTL discovery in COMB dataset, significance thresholds were determined using a stringent threshold using Bonferroni multiple testing correction; genome-wide significance thresholds were obtained by dividing the nominal significance threshold of 0.05 by the total numbers of SNP (38,545) included in the analysis. The p-value after Bonferroni multiple testing corrections was 1.297e-06 (i.e. –log_10_(p-value) = 5.89).

If a stringent threshold is used in validation studies then few validated association will be recorded while too low threshold will results in many false positive associations. Therefore, we used chromosome-wise significant threshold in the reference population (REF). While a threshold of P <0.05 was used for the validation population (VAL) following Kemper et al. [10].

The false discovery rate (FDR) is the ratio of the expected significant associations to the actual number of significant associations. FDR was calculated following Benjamini and Hochberg [11] as FDR = mP/S, where m is the number of markers tested, P is the significance threshold (p-value) and S is the number of markers with significant associations.

### Demarking the QTL Region

Normally, multiple SNPs in the vicinity of a QTL are expected to yield significant results in a SNP-by-SNP analysis. This is because sets of SNPs that are physically located near the causal factor will tend to be in linkage disequilibrium; this effect declines with genetic distance and also depends on MAFs. The software ChromoScan [12] was used to identify the chromosomal region harboring the QTL. Using a compound Poisson process, the scan statistical methodology in ChromoScan takes account of the complex distribution of genome variation in the identification of chromosomal regions with significant clusters of SNP-trait associations. The interval which harbors the most significantly associated SNP was chosen as the putative QTL interval.

## Results

### Follow-up study

**Figure 1:**
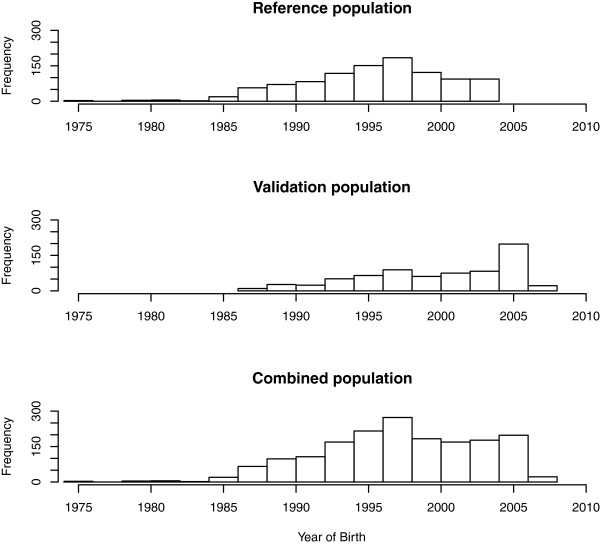
Distribution of birth-years of the individuals from three datasets.

The number of genome-wide significant SNPs for three datasets, full data (COMB), reference dataset (REF) and validation set (VAL) are presented in Table 2. Analyzing COMB which had a sample size twice that of the other two subsets detected far more significant associations than analyzing the two smaller datasets individually when genome-wide multiple testing correction was done. The number of significant SNPs in REF was about four times higher compared to VAL despite both having similar sample sizes for some traits. For example, DCSL had highest number of significant SNPs for all the three datasets (Table 2). DCEL also had relatively more phenotypic records than many of the other traits analyzed here (Table 1). The distributions of birth-years of individuals in three datasets are presented in Figure 1. All the animals included in the REF dataset were born in or before the year 2004, while 36% of the animal in the VAL dataset were born after the year 2004.

**Table 2:** Number of SNP-trait combinations showed genome-wide significant association for three datasets

**Trait**^**#**^	**COMB**	**REF**	**VAL**
BI	37	11	3
CI	46	7	0
DCEF	11	5	1
DCEL	54	14	6
DSBF	1	1	0
DSBL	7	2	2
DCSF	24	3	1
DCSL	123	29	9
MCEF	38	11	0
MCEL	2	0	0
MSBF	58	3	1
MSBL	4	5	0
MCSF	16	9	0
MCSL	3	1	1
Total	424(284)	101(73)	24(18)

Figures in parenthesis are number of unique SNPs showing association with one or more traits.

^#^Prefixes: D = direct effect; M = maternal effect. Calving traits: BI = birth index; SB = stillbirth; CE = calving ease; CS = calf survival; CI = calving index. Suffixes: F = first lactation; L = later lactation.

### Validation of SNP associations

In the REF data set 469 unique significant SNP were found at chromosome wide significant threshold of which 321 could be confirmed in the VAL dataset at P < 0.05. The FDR was 7.3% in the validation population. This suggests that there will be about 296 (i.e. 321 validated SNP x 7.3% FDR = 23.4 false positive SNP) real SNP with validated association.

### Mapping of QTL affecting calving traits using the combined dataset

**Figure 2:**
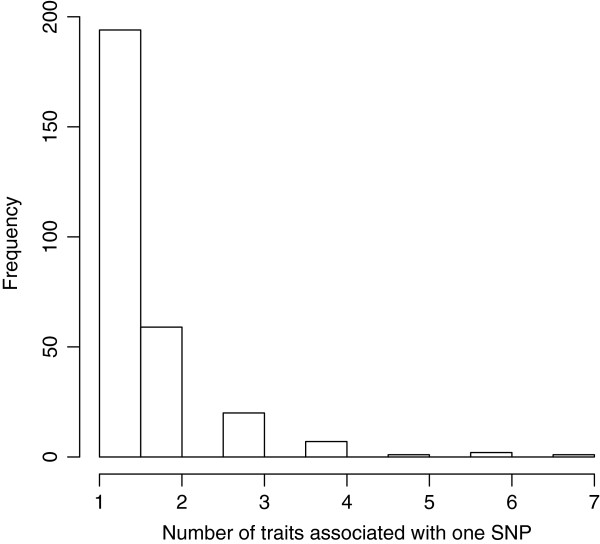
The number of SNP markers (y-axis) significantly associated to one or more traits (x-axis).

A total of 424 SNP-trait combinations on 22 chromosomes showed genome-wide significant association involving 284 unique SNPs in the COMB data analyses. Out of these total 424 combinations, 220 combinations were for the direct calving traits and 121 were for maternal calving traits. The remaining associations involved the two indices (BI and CI). There were 37 and 46 genome-wide significant associated SNPs for BI and CI. About half of the SNPs showing significant association were associated with only one calving trait, while the remaining half showed associations with multiple traits (Figure 2). We observed genome-wide significant associations for all the 14 traits analyzed in this study. The highest number was 123 SNPs showing association with DCSL followed by MSBF (58), CI (46), MCEF (38) and BI (37). The chromosomes with large numbers of significant SNP-trait associations were BTA18 (89) followed by BTA13 (50), BTA9 (45), BTA19 (37), BTA3 (35) and BTA1 (28). No SNPs on seven chromosomes (BTA2, 14, 16, 24, 27, 28 and 29) showed genome-wide significant association with any of the 14 calving traits. One SNP (SS86324977) on BTA18 was significant for 7 traits and was the most significant SNP (lowest p-value) for all these 7 traits. This SNP is located in an intron of the sialic acid binding Ig-like lectin-5(SIGLEC5) gene, and has been earlier identified to affect many calving traits [13].

**Figure 3:**
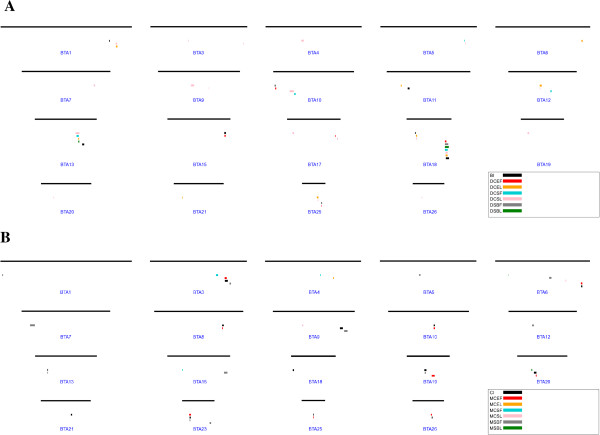
The QTL regions affecting calving traits in Nordic Holstein population from the analyses combined dataset. A Direct calving traits; B Maternal Calving traits.

Additional file 1 Table S1 presents the QTL regions demarked where most significant SNP was located where ChromoScan gave a significant interval based on the p-values of the markers in that interval. There were a total of 91 QTL listed in the Additional file 1 Table S1. The QTL regions were also plotted in Figure 3. The QTL intervals ranged from 0.36 to 5.94 Mbp.

## Discussion

### Follow-up study

Here we have compared two strategies of how to analyze a follow-up GWAS study in cattle when new data is accumulated. We found that studying the VAL dataset as independent clearly under-performs and missed to detect most of the QTL if multiple testing criteria are applied for significant threshold. The power of this approach was only 6% compared to analyzing COMB. There were several reasons for such low power. As we used a very stringent level of significance, many of the p-values in VAL and REF did not reach the threshold. For example, the numbers of genome-wide significant SNP-trait associations detected for the trait DCSL were 123, 29 and 9 for COMB, REF and VAL datasets respectively (Table 2). One reason for difference in power for three datasets was the difference in sample sizes. The three datasets had the same number of SNPs tested but had different sample sizes (3559 for COMB; 2034 for REF and 1525 for VAL). It was expected that at the nominal p-value of 0.05 after multiple testing correction, the COMB dataset (with the largest sample size) will not have higher type-I error rate than the two sub datasets. Therefore, the additional QTL identified by COMB are likely to be true ones and not false positives.

One of the critical factors that influence the power of detection of a QTL is how much of the phenotypic variance is explained by a QTL. The higher the QTL heritability, the chance of detecting the QTL increases. We observed marked difference in power in the two subsets of data (REF and VAL) even though they had nearly the same number of records for some traits e.g. BI (Table 1 and 2). However, this observation may be explained by differences in individuals in the REF and VAL datasets. First, the phenotypes used here were breeding values of bulls which were estimated from their daughters’ performance. The bulls in REF were born in or before the year 2004 while 36% of the bulls in the VAL were born after 2004 and therefore the STBVs for the sire in REF dataset had presumably more accurate breeding values because these were based on a higher number of daughters compared to the ‘younger’ bulls. The higher the accuracy of the breeding value the closer it is to the genotypic value of an individual. Another reason could be the family structure of the REF and VAL datasets. The REF population was from an earlier within-family linkage study for QTL mapping [14]. Therefore, in the REF we expect to have larger half sib families. For the QTL segregating in those large grandsire families, one will expect that these have relatively higher power to be detected due to within family linkage information. In the VAL, there may be a higher number of QTL segregating but with less power to be detected due to smaller family sizes.

### Comparison to the previously reported QTL studies

Many QTL studies on birth related traits in cattle have been performed using linkage analyses with microsatellite markers, which make direct comparisons of the results even more difficult due to the large confidence intervals for QTL positions. However, even though precise QTL location cannot be compared, QTL detected in the vicinity on the same chromosomal position in other populations strengthen the result of QTL being real. Direct comparison between data obtained in this study and those from other previous linkage-based studies is hindered by the fact that locations in the linkage map given in centiMorgan (**cM**), do not necessarily reflect the same physical location on the genome derived from different assemblies. Therefore, the physical locations of the reported markers from previous studies were mapped to the University of Maryland assembly UMD3.1, which enables direct comparison. In the discussion, we focus on the nine chromosomes on which QTL were detected for more than one trait. This discussion is in reference to the results from the COMB dataset which had higher power to detect QTL compare to the other two subsets (REF and VAL). However, we have also discussed if we could validate those previously reported QTL in our validation analysis. It should be noted that the conclusions of Thomasen et al. [14] were based on analyses of part of the population constituting the REF dataset. Their marker data were microsatellites types, but part of the phenotypic information overlaps the phenotypic information used in the current study.

The most significant QTL across all chromosomes was located on BTA18. It was associated with seven different calving traits. This QTL was detected previously in Danish Holstein [14], German Holstein [15] and in Finnish Ayrshire cattle [16]. The most significant SNP, SS86324977, is located in an intron of the sialic acid binding Ig-like lectin-5 gene, was previously identified as affecting many calving traits [13]. In this study 36 unique SNPs could be confirmed in the VAL dataset compared to the REF reflecting different calving traits on chromosome 18 (Table 3).

**Table 3:** Chromosome-wise number of significant SNPs in reference population (REF) and the confirmed SNP in the validation population (VAL)

**chromosome**	^**1**^**Unique significant SNPs in REF dataset**	^**2**^**Confirmed in VAL dataset**	^**3**^**Number of confirmed SNPs within boundary of QTL in COMB dataset**
1	22	15	7
2	3	2	-
3	41	31	6
4	20	8	3
5	7	5	2
6	52	22	5
7	8	5	1
8	8	5	2
9	27	19	9
10	19	10	4
11	8	5	2
12	12	6	4
13	34	17	10
14	4	2	-
15	25	15	2
16	9	6	-
17	6	4	-
18	99	36	19
19	62	39	15
20	27	9	5
21	5	1	-
22	1	1	-
23	39	20	4
24	3	1	-
25	60	28	9
26	11	6	3
28	11	2	-
29	3	-	-

^1^Number of unique SNPs in the REF dataset those were significant chromosome wide level.

^2^Confirmed in the VAL dataset at P < 0.05.

^3^Any of the confirmed SNPs that fell in the boundaries of QTL defined for the COMB dataset of any trait presented in Table 4.

**Table 4:** QTL regions defined form the analysis of the combined dataset (COMB). The positions are as per UMD3.1

**chromosome**	**Position (Bp)**		**Traits**^**#**^
	**Lower Boundary**	**Upper boundary**	
1	138993085	140524195	DCEL,DCSL
3	83093150	85629524	MCSF
4	44952454	47737653	DCSL
6	108284991	109951981	CI,DCEL,MCEF
8	86613020	88464533	CI,MCEF
9	92300111	96298532	MSBF
9	37914265	39339429	MCSL
11	18221603	19125116	DCEL
13	16953393	17383654	CI
13	55613243	60468287	DCEL,DSBL,DCSF,DCSL
15	77169139	81574949	BI,DCEF,MSBF
18	56642741	62221442	BI,DCEF,DCEL,DSBF,DSBL,DCSF, DCSL
18	13839646	15225943	DCEL,DCSL
20	17946080	18537789	DCSL
20	26602523	27928617	MCEF
23	12027791	14063300	CI,MCEF,MSBF
25	27454679	30187316	DCEL,DCSL,
26	14934474	15604631	DCSL

^#^Prefixes: D = direct effect; M = maternal effect. Calving traits: BI = birth index; SB = stillbirth; CE = calving ease; CS = calf survival; CI = calving index. Suffixes: F = first lactation; L = later lactation.

On BTA1, QTL were detected for BI, CI, DCSL, and DCEL. QTL regions for DCSL and DCEL overlapped. Supportive evidence for these QTL was not found in the literature. Fifteen significant SNPs from REF were confirmed in the VAL dataset for different traits. Confirmed SNPs were found most pronounced in the same regions as DCSL and DCEL in the COMB dataset.

On BTA3, QTL were detected for CI, DCSC, MCEF, MSBF, and MCSF. QTL regions for MCEF and CI overlapped in chromosomal locations. Thomasen et al. [14] identified a QTL for direct stillbirth in the Danish Holstein cattle population, in the vicinity of the QTL detected for CI in this study. CI represents the maternal effect of calving traits and does not include direct stillbirth in the calculation. Therefore, this QTL might have effect on both direct and maternal calving traits. In the confirmation 31 SNPs were confirmed, the most confirmed SNPs were found in the regions of MCEF and CI in the COMB dataset.

A total of six QTL were identified on BTA10. The QTL for CI and MCEF as well as for BI and DCEF showed overlap, whereas the QTL for DCSF and DCSL did not show overlap to any of the other QTL detected on this chromosome. A QTL for maternal calving difficulty was detected in a previous study in the Danish Holstein cattle by Thomasen et al. [14], but at different chromosomal location. Seidenspinner et al. [17] detected a QTL in the German Holstein population for maternal dystocia at a different location than observed in this study. Ten SNPs were confirmed in the REF and VAL dataset, primarily in the region of DCSF and DCSL but also in the region of DCEF.

On BTA13, QTL for BI, CI, DCEL, DSBL, DCSF, DCSL, and MSBF were detected in this study. A QTL for calving difficulties was detected in Swedish Holstein cattle on the same chromosome [18] but at a different location compared to our result. In the German Holstein population a QTL for direct dystocia was detected [17]. Confirmation was observed for 17 SNPs. The confirmation was mostly pronounced in the region of DCEL, DSBL, DCSF and DCSL. The list of the genes in the region (55,613,243bp-60,300,299bp) from http://www.ensembl.org/biomart/ database is presented in Additional file 2 Table S2. However, further studies are needed to point out any obvious candidate gene.

On BTA19, four QTL were detected for CI, DCSL, MCEF, and MSBF. The QTL regions for CI and MSBF show overlap. MSBF is included in the calving index; thereby the QTL effect on CI might be mediated through stillbirth. A QTL was detected for direct calf size in the Danish Holstein population on BTA19 covering a large confidence interval [14] and the QTL for MSBF detected in this study was within this confidence interval. In the German Holstein cattle population QTL for direct and maternal dystocia had been found [17]. These QTL did not show overlap with the QTL detected in the present study. A QTL for maternal dystocia was detected by Seidenspinner et al. [17] in the vicinity of the QTL for DCEF detected in our study. Thirty-six SNP associations on this chromosome from the REF could be validated in the VAL population. The majority of the validated SNPs were in the region of MCEF, MSBF and CI.

On BTA20, four QTL were detected, one for each of CI, DCSL, MCEF and MSBL. The QTL for CI and MCEF show overlap. In a previous study using the Danish Holstein cattle a QTL for direct calving size was detected [14]. This QTL did not overlap with the QTL detected in our study. Also, in Norwegian Red cattle a QTL was detected for direct stillbirth [19], however this QTL did not overlap with any of the QTL detected on BTA20 in our study. Nine SNPs were confirmed between the REF and the VAL dataset. The most confirmed SNPs were found in the region of CI and MCEF.

One QTL each was detected for CI and MCEF, whereas for MSBF two QTL were detected on BTA23. The QTL for CI, MCEF, and MSBF showed overlap in the region 11589692bp-14063300bp. Within this region 22 genes (http://www.ensembl.org/biomart) have been annotated but again there is a need for further studies to point out an obvious candidate gene (Additional file 3 Table S3). The second QTL for MSBF was located at the other end of the chromosome. In the German Holstein cattle population a QTL was detected on BTA23 for maternal and direct stillbirth as well as maternal and direct dystocia [17]. These QTL were not in the vicinity of the QTL detected in the present study. We could confirm 20 SNPs on this chromosome, the most confirmed SNPs were found in the region of CI, MCEF and MSBF.

In total six QTL were detected on BTA25, one each for BI, CI, DCEF, DCEL, DCSL, and MCEF. The QTL regions overlapped between CI and MCEF, between DCEL and DCSL, and between BI and DCEF. In the Danish Holstein population two QTL had been detected previously for direct calving size and direct calving difficulty within the same marker brackets on BTA25 [14]. Though these QTL did not overlap with any of the QTL detected in this study, they were in the vicinity of QTL detected for DCEL and DCSL. A QTL for maternal dystocia was detected in the German Holstein cattle [17], but at the other end of the chromosome. We could confirm 28 SNPs spanning over all the regions for the QTL discovered in the combined dataset.

## Conclusion

The relative merit of analyzing a combined datasets in association study to analyzing only new accumulated data in dairy cattle population was evaluated. The analyses of the VAL population as an independent new dataset clearly under-performs and failed to detect most of the QTL detected in the previous study if stringent multiple testing correction is applied. The larger combined datasets analyses showed higher power.

The follow-up study for GWAS will depend on the aim of the study. If the aim is to discover novel QTL, analyses of the COMB dataset is recommended, while in case of identification of the causal mutation underlying a QTL, validation of the discovered SNPs are necessary to avoid following a false positive.

We also identified several novel QTL affecting the calving performance in dairy cattle.

## Competing interests

The authors declare that they have no competing interests.

## Authors’ contributions

JKH, GS- Performed the analysis, discussed the results and wrote the manuscript. BG, MSL contributed to the planning and discussion of the results. All authors read and agreed on the contents of the manuscript.

## Supplementary Material

Additional file 1 **Table S1.** Chromosomal location of the QTL for calving traits. Click here for file

Additional file 2 **Table S2.** List of genes located in the QTL region on BTA13. Click here for file

Additional file 3 **Table S3.** List of genes located in the QTL region on BTA23. Click here for file

## References

[B1] Witte JS Elston RC Cardon LR On the relative sample size required for multiple comparisons Stat Med 2000 19 369 372 10.1002/(SICI)1097-0258(20000215)19:3<369::AID-SIM335>3.0.CO;2-N 10649302

[B2] Thomas DC Haile RW Duggan D Recent developments in genome wide association scans: a workshop summary and review Am J Hum Genet 2005 77 337 345 10.1086/432962 16080110 PMC1226200

[B3] NCI-NHGRI Working Group on Replication in Association Studies Replicating genotype-phenotype association Nature 2007 447 655 660 10.1038/447655a 17554299

[B4] Sørensen AC Sørensen MK Berg P Inbreeding in Danish Dairy Cattle Breeds J Dairy Sci 2005 88 1865 1872 10.3168/jds.S0022-0302(05)72861-7 15829680

[B5] Guldbrandtsen B Höglund JK Lund MS Sahana G Analyzing follow-up studies to genome wide association studies Proceedings of the Ninth World Congress on Genetics Applied to Livestock Production: 1-6 August 2010 CD-ROM communication no. 0765, Leipzig, Germany

[B6] Sahana G Guldbrandtsen B Lund MS Genome-wide association study for calving traits in Danish and Swedish Holstein cattle J Dairy Sci 2011 94 479 486 10.3168/jds.2010-3381 21183059

[B7] Danish Cattle Federation Principles of Danish Cattle Breeding 2006 http://www.lr.dk/kvaeg/diverse/principles.pdf

[B8] Boelling D Sander Nielsen U Pösö J Eriksson J-Å Aamand GP Genetic evaluation of calving traits in Denmark, Finland, and Sweden Interbull Bull 2007 37 179 184

[B9] Pedersen J Sørensen MK Toivonen M Eriksson J Aamand GP Report on economic basis for a Nordic total merit index http://www.nordicebv.info/NR/rdonlyres/BFC1E284-4DC9-4E7C-96F6-F7A6EA0CDF6B/0/NAV_TMI_Light_report.pdf 10.3168/jds.2013-7694 25306270

[B10] Kemper KE Emery DL Bishop SC Oddy H Hayes BJ Dominik S Henshall JM Goddard ME The distribution of SNP marker effects for faecal worm egg count in sheep, and the feasibility of using these markers to predict genetic merit for resistance to worm infections Genet Res 2011 93 203 219 10.1017/S0016672311000097 24725775

[B11] Benjamini Y Hochberg Y Controlling the false discovery rate: a practical and powerful approach to multiple testing Journal of the Royal Statistical Society, Series B (Methodological) 1995 57 289 300

[B12] Sun YV Jacobsen DM Kardia SLR ChromoScan: A Scan Statistic Application for Identifying Chromosomal Regions in Genomic Studies Bioinformatics 2006 22 2945 2947 10.1093/bioinformatics/btl503 17032677

[B13] Cole JB Van Raden PM O’Connel JR Van Tassel CP Sonstegard TS Schnabel RD Taylor JF Wiggans GR Distribution and location of genetic effects for dairy traits J Dairy Sci 2009 92 2931 2946 10.3168/jds.2008-1762 19448026

[B14] Thomasen JR Guldbrandtsen B Sørensen P Thomsen B Lund MS Quantitattive trait loci affecting calving traits in Danish Holstein cattle J Dairy Sci 2008 91 2098 2105 10.3168/jds.2007-0602 18420641

[B15] Brand B Baes C Mayer M Reinsch N Seidenspinner T Thaller G Kühn C Quantitative trait loci mapping of calving and conformation traits on Bos *Taurus* autosome 18 in the German Holstein population J Diary Sci 2010 93 1205 1215 10.3168/jds.2009-2553 20172241

[B16] Schulman NF Sahana G Lund MS Viitala SM VIlkki JH Quantitative trait loci for fertility traits in Finnish Ayrshire cattle Genet Sel Evol 2008 40 195 214 18298935 10.1186/1297-9686-40-2-195 PMC2674925

[B17] Seidenspinner T Bennewitz J Reinhardt F Thaller G Need for sharp phenotypes in QTL detection for calving traits in dairy cattle J Anim Breed Genet 2009 126 455 462 10.1111/j.1439-0388.2009.00804.x 19912419

[B18] Holmberg M Andersson-Eklund L Quantitative Trait Loci affecting Fertility and Calving Traits in Swedish Dairy Cattle J Dairy Sci 2006 89 3664 3671 10.3168/jds.S0022-0302(06)72406-7 16899702

[B19] Olsen HG Hayes BJ Kent MP Nome T Svendsen M Lien S A genome wide association study for QTL affecting direct and maternal effects of stillbirth and dystocia in cattle Anim Genet 2010 41 273 280 10.1111/j.1365-2052.2009.01998.x 19968646

